# PD-L1 and HER2 Expression in Gastroesophageal Cancer: a Matched Case Control Study

**DOI:** 10.1007/s12253-020-00814-2

**Published:** 2020-05-05

**Authors:** Andrea Beer, Hossein Taghizadeh, Ana-Iris Schiefer, Hannah C. Puhr, Alexander K. Karner, Gerd Jomrich, Sebastian F. Schoppmann, Renate Kain, Matthias Preusser, Aysegül Ilhan-Mutlu

**Affiliations:** 1 Department of Pathology, Medical University of Vienna, Wien, Austria; 2 Comprehensive Cancer Center Vienna, Upper GI Tumors Unit, Medical University of Vienna, Wien, Austria; 3 Department of Medicine I, Clinical Division of Oncology, Upper Gastrointestinal Tumors Unit, Medical University of Vienna, Wien, Austria; 4 Department of Surgery, Medical University of Vienna, Wien, Austria

**Keywords:** Gastroesophageal tumor, Esophageal tumor, Gastroesophageal junction tumor, Gastric tumor, PD-L1, HER2, Immunotherapy, TPS, CPS

## Abstract

Immunotherapy with check-point inhibitors serves as a promising treatment strategy in patients with upper gastrointestinal (GI) tumors. Human epidermal growth factor receptor 2 (HER2) is the only identified therapeutic target in upper GI tumors, whose potential interaction with programmed death-ligand 1 (PD-L1) is unknown. The aim of this study was the investigation of PD-L1 and HER2 in upper GI tumors. We retrospectively identified patients with HER2 positive gastroesophageal cancers and matched them with a HER2 negative group. We investigated the tumor specimens for HER2 status and PD-L1 expression, with the following assessments being performed: i) staining of tumor cells in terms of tumor proportion score (TPS), ii) staining for tumor-associated immune cells (TAIs), iii) interface pattern and iv) combined positive score (CPS). Both HER2 positive and negative group consisted of 59 patients. Expression of PD-L1 in TAIs and interface pattern were associated with a favorable outcome (*p* = 0.02, HR = 0.8; *p* = 0.04, HR = 0.39; respectively) in patients with localized disease, whereas TPS was associated with an unfavorable outcome in patients with advanced tumor (p = 0.02, HR = 1.4). These effects were HER2 independent. PD-L1 expression in its different assessment is equally observed in HER2 positive and negative patients. Future studies will show whether dual inhibition of HER2 and PD-L1 improves survival of this selected patient population.

**Electronic supplementary material:**

The online version of this article (10.1007/s12253-020-00814-2) contains supplementary material, which is available to authorized users.

## Introduction

Gastric cancer is the fourth most commonly diagnosed cancer and the second most common cause of cancer related deaths worldwide [1]. Most patients present with inoperable advanced or metastatic disease requiring palliative treatment. Five-year survival for advanced or metastatic gastric, gastroesophageal junction (GEJ) or esophageal cancer (together upper GI tumors) is approximately 5–20%, with a median overall survival (OS) of about 1 year. There is currently not a single well-established standard of care, but fluoropyrimidine-based and platinum-based combinations with or without a third drug (usually taxane or anthracycline) are the most commonly used combinations in Europe and the USA [1].

Recently, advances in technology and high-throughput analysis have improved our understanding of the genetic basis of gastric cancer. To provide a roadmap for patient stratification and trials of targeted therapies, the Cancer Genome Atlas (TCGA) Research Network has characterized 295 primary gastric adenocarcinomas and proposed a new classification of four different tumor subtypes of Epstein-Barr virus positive, microsatellite instable (MSI), genomically stable and chromosomal instable subtypes [2]. Epstein-Barr virus positive (around 9%) gastric cancers are generally characterized by some distinct genetic features including increased number of tumor infiltrating lymphocytes and programmed cell death-ligand 1 (PD-L1) positivity [3, 4].

PD-L1 is a 40-kDA transmembrane protein that is activated in many cancer types and thereby leads to an immunosuppressive tumor microenvironment. Thus, inhibition of PD-L1 and its receptor PD-1 have been intensively studied as novel treatment concepts in various cancer diseases including malignant melanoma, lung cancer and renal cell carcinoma [5]. A phase Ib and a follow-on phase II trial showed a promising overall response when treating patients with PD-L1 positive gastroesophageal cancer in a salvage setting with the anti-PD-1 antibody pembrolizumab [6, 7]. Based on these trials, the FDA approved pembrolizumab for the treatment of PD-L1 positive gastroesophageal cancer in later lines. Furthermore, a recent phase III trial in already extendedly treated patients with gastric cancer demonstrated an efficacy with another PD-1 inhibitor, nivolumab, in an Asian population, which has led to its approval as a salvage treatment in Japan [8].

Targeted therapies are introduced for clinical use in patients with advanced upper GI tumors. Up to 20% of gastric tumors overexpress human epidermal growth receptor 2 (HER2) [9–11]. The pivotal ToGA (Trastuzumab for Gastric Cancer) trial was the first randomized, prospective, multicenter phase III trial to study the efficacy of first-line trastuzumab (a monoclonal antibody against HER2) in patients with HER2 positive advanced upper GI tumors [9]. On the basis of this study, trastuzumab in combination with cisplatin and a fluoropyrimidine has been approved for first-line treatment of advanced HER2-positive upper GI tumors.

There exists varying information on the expression of HER2 and the prognosis of patients with upper GI tumors. On the one hand, poor outcome and fast progression are often described [12–14], on the other hand comparable survival times with HER2 negative patients were also shown [15]. Recently, Gu et al. performed a meta-analysis of the prognosis of HER2 positive patients, who were diagnosed according to ToGA criteria, where no difference in survival was observed between negative and positive patients [16]. Our own observation demonstrated an overall survival of 21 months for patients with advanced gastroesophageal adenocarcinoma, which is remarkably longer than that observed in the ToGa trial [17].

Although trastuzumab extends the survival of HER2 positive patients, these patients typically develop treatment resistance, and second-line treatment options are limited. Attempts to use other HER2 targeted therapies failed to demonstrate any survival benefit both in first- [18, 19] and second-line settings [20, 21]. HER2 positive gastroesophageal carcinoma patients are for the most part excluded from clinical trials investigating immunotherapy drugs since the expression might bias the results due to the distinct biological character of this group. Moreover, involvement of the PD-L1 status in HER2 positive patients or potential interactions are not clearly known. The aim of this study was to test the expression and distribution level of PD-L1 in HER2 positive gastroesophageal cancers and compare these values against the matched HER2 negative samples.

## Materials and Methods

### Patient Collection

Patients with biopsy- or resection-confirmed diagnosis of gastroesophageal cancer and older than 18 years of age were selected by a comprehensive search of the chart data of the Medical University of Vienna. Cases with known HER2 positive status were recruited and matched with HER2 negative ones based on tumor type (adenocarcinoma or squamous cell carcinoma), staging at the time of initial disease onset (localized or advanced/metastasized) and gender. Demographic, clinical, pathological and survival parameters were retrieved from chart data as well.

Adenocarcinomas were subdivided according to Laurén classification into intestinal, diffuse and mixed subtypes [22] and according to WHO 2019 classification into tubular, papillary, poorly cohesive (signet ring cell type and not signet ring cell type), mucinous and mixed subtypes. Patients were followed up until death as documented in the hospital records or until they were lost to follow-up.

### Treatment Schedule and Response Evaluation

Patients without any signs of distant metastasis preferably received neoadjuvant treatment, which was followed by surgical resection of the tumor. After an adjuvant chemotherapy period, routine control visits with computed tomography (CT) scans every 3 months were performed.

Patients with typical signs of distant metastasis underwent palliative chemotherapy. If the tumor was HER2 positive, trastuzumab was added to the treatment schedule. Trastuzumab was administered by intravenous infusion at a dose of 8 mg/kg on day 1 of the first cycle, followed by 6 mg/kg every 3 weeks until progression of the disease, the occurrence of unacceptable toxicity, or the patient’s refusal. After administration of 3 cycles of chemotherapy or trastuzumab containing treatment, the size of the tumor was investigated by CT imaging, and the tumor response was classified according to RECIST [23]. Patients with typical signs of progressive disease or recurrence were subjected to second-line treatment or palliative chemotherapy, respectively. Surgical resection of the primary tumor or metastases was performed individually for some patients. If there was no progression of the disease during or after chemotherapy, patients received trastuzumab as a maintenance treatment until progression.

### HER2 Analysis

Gastroesophageal adenocarcinomas were routinely tested for HER2 status with immunohistochemistry (790–2991, Ventana). In cases with equivocal results (2+) the samples were re-examined with chromogenic in situ hybridization (CISH, 800–4422, Ventana) or fluorescence in situ hybridization (FISH, 06 N46–036, Abbott-PathVysion). In CISH and FISH the HER2 gene copy number and centromere enumerator probe 17 (CEP17) were investigated. The assignment to HER2 positive or negative was based on the study of Hofmann and colleagues [13]. Patients were allocated to receive trastuzumab if their tumor samples were scored as 3+ in immunohistochemistry, or in case of 2+, if they were amplified in CISH or FISH (HER2:CEP17 ratio ≥ 2).

The HER2 staining results, which were obtained from the hospital chart data, were re-evaluated in the frame of this study for both (HER2 positive and negative) groups.

### Evaluation of the PD-L1 Immunostaining

One representative section of each surgical tumor resection or biopsy specimen was stained with antibodies against PD-L1 (M3653, Dako). The immunoreactivity of PD-L1 was evaluated according to the percentage of membranous (complete or incomplete) positively stained tumor cells (Tumor proportion score (TPS)) and the percentage of positively stained tumor associated immune cells (TAIs), i.e. all immune cell subtypes (categorization into 0%, 1%, 3%, 5%, 10%, 15%, 20%, 30%, etc. in both). Staining intensity was not considered. Specimens in which PD-L1 staining was observed in ≥1% of tumor cells or immune cells were considered PD-L1 positive.

Additionally, the combined positive score (CPS) and the interface pattern were evaluated. CPS was calculated by dividing the number of PD-L1 positive tumor cells, lymphocytes and histiocytes by the total number of vital tumor cells and then multiplying the result by 100. The interface pattern was described by Muro K et al. as a band of PD-L1-positive cells (mainly mononuclear inflammatory cells) at the interface between confluent areas of neoplastic cells and adjacent stroma [6]. We evaluated the presence of this pattern dichotomously (yes/no). All analyses were performed independently by two experienced pathologists (A.B., AI.S) and in case of differing results a consensus was reached together.

### Statistical Analyses

Student’s t test or Mann-Whitney U-Test were used for the comparison of mean values for the parametric and non-parametric distribution. Chi-Square Test was utilized for the analysis of the distribution of the dichotomized variables. ANOVA tests were applied where multiple testing was necessary. Differences between tumor marker values before and after chemotherapy were calculated using paired t-test. For patients without an event (death) the cut-off was the date that they were last known to be alive. OS was calculated from the date of initial diagnosis of gastroesophageal cancer to the death of the patient or the patient’s last follow-up date. PFS was measured from the first date the first-line systemic anti-tumor treatment was administered to the date of disease progression confirmed by CT scans. Kaplan-Meier survival estimates with log rank test and Cox regression analyses of OS and PFS were performed. Cox regression analysis was used to correlate the following parameters with outcome: Gender, age, carcinoma type (squamous cell or adenocarcinoma), tumor location, Laurén classification, family history of any cancer, family history of gastrointestinal cancer, second oncology, nicotine consumption, number of metastatic sites, location of the metastases and grading. Pearson’s correlation coefficient was employed for the correlation analysis between different scores. Two-tailed *p* values of 0.05 or less were considered to be significant. Tests for the expression values of HER2 and PD-L1 were corrected for the biopsy or surgical specimen status. All statistics were calculated using the statistical package for the social sciences (SPSS) 24.0 software (SPSS Inc., Chicago, IL, USA). GraphPad Prism Version 8 (GraphPad Software Inc.; San Diego, CA, USA), SPSS and Microsoft Power Point were used for creation of the figures.

### Ethical Approval and Ethical Standards

This study was approved by the local ethic committee (ethics committee of the Medical University of Vienna, Reference number: 2267/2016) in accordance with the Helsinki Declaration of 1975. All methods were carried out in accordance with these guidelines and regulations.

### Informed Consent

No informed consent is necessary in the scope of this study, since the specimen were analyzed retrospectively and already belonged to the Medical University of Vienna at the time of the analyses. This approach was in accordance with the guidelines of the ethics committee of the Medical University of Vienna.

## Results

### Demographics

We identified 59 HER2 positive and 59 matched HER2 negative patients, who underwent tumor biopsy or resection in the years 1997 to 2017 at our institution. Patient demographics are shown in Table 1.

**Table 1:** Patient demographics and baseline characteristics

			HER2 positive (*n* = 59)	HER2 negative (n = 59)	*p*
Age (Years/Range)			62 (35–91)	66 (37–88)	n.s.
Women (n/percentage)			11 (19%)	11 (19%)	n.s.
Positive family history for oncological diseases (yes/percentage)			13 (22%)	14 (24%)	n.s.
Positive family history for gastrointestinal malignancies (yes/percentage)			2 (3%)	4 (7%)	n.s.
Second tumor disease (yes/percentage)			12 (20%)	5 (8%)	n.s.
Nicotin abusus (yes/percentage)			23 (39%)	21 (36%)	n.s.
Primary tumor side					n.s.
	Stomach (yes/percentage)		8 (14%)	6 (10%)	
	GEJ (yes/percentage)		48 (81%)	50 (85%)	
	Esophagus (yes/percentage)		3 (5%)	3 (5%)	
Histology					n.s.
	Adenocarcinoma (yes/percentage)		57 (97%)	57 (97%)	
	SCC (yes/percentage)		2 (3%)	2 (3%)	
WHO 2019 Classification					n.s.
	Tubulary		26 (44%)	19 (32%)	
	Papillary		5 (8%)	2 (3%)	
	Poorly cohesive - signet ring cell type		1 (2%)	5 (8%)	
	Poorly cohesive - non signet ring cell type		4 (7%)	13 (22%)	
	Mucinous		0	4 (7%)	
	Mixed types		21 (37%)	13 (22%)	
	Squamous		2 (3%)	2 (3%)	
	Adenosquamous		0	1 (2%)	
Lauren Classification					**0.001**
	Diffuse (yes/percentage)		5 (8%)	27 (46%)	
	Intestinal (yes/percentage)		42 (71%)	17 (29%)	
	Mixed (yes/percentage)		11 (19%)	11 (19%)	
Advanced disease (yes/percentage)			20 (34%)	20 (34%)	n.s.
Number of metastatic sites per patient (n/range)			1 (1–3)	1 (1–3)	n.s.
	1		13 (22%)	9 (15%)	
	2		5 (8%)	5 (8%)	
	3		2 (3%)	1 (3%)	
Metastatic sites					n.s.
	Liver (n/percentage)		11 (19%)	9 (15%)	
	Peritoneum (n/percentage)		6 (10%)	3 (5%)	
	Lymphnode (n/percentage)		2 (3%)	1 (2%)	
	Lung (n/percentage)		6 (10%)	3 (5%)	
	Bones (n/percentage)		1 (2%)	3 (5%)	
	Muscles (n/percentage)		1 (2%)	2 (3%)	
	Omentum (n/percentage)		0	1 (2%)	
Tumor tissue type					n.s.
	Biopsy (n/percentage)		24 (41%)	16 (27%)	
	Resection (n/percentage)		35 (60%)	43 (73%)	
Tumor Grade					**0.006**
	I (n/percentage)		0	2 (3%)	
	II (n/percentage)		31 (53%)	18 (31%)	
	III (n/percentage)		19 (32%)	36 (61%)	
TNM Classification					
	T				n.s.
		1 (n/percentage)	7 (12%)	5 (8%)	
		2 (n/percentage)	6 (10%)	8 (14%)	
		3 (n/percentage)	24 (41%)	29 (49%)	
		4 (n/percentage)	0	1 (2%)	
	N				n.s.
		0 (n/percentage)	14 (24%)	7 (12%)	
		1 (n/percentage)	17 (29%)	27 (46%)	
		2 (n/percentage)	7 (12%)	9 (15%)	
		3 (n/percentage)	1 (2%)	3 (5%)	
	L				n.s.
		0 (n/percentage)	9 (15%)	3 (5%)	
		1 (n/percentage)	15 (25%)	14 (24%)	
	V				**0.03**
		0 (n/percentage)	16 (27%)	8 (14%)	
		1 (n/percentage)	6 (10%)	12 (20%)	
	R				n.s.
		0 (n/percentage)	23 (40%)	19 (32%)	
		1 (n/percentage)	4 (7%)	4 (7%)	
Gastrectomy (yes/percentage)					
	Palliative (yes/percentage)		9 (45%)	3 (15%)	n.s.
	Curative		37 (95%)	39 (100%)	n.s.

n, number; HER2, human epidermal growth factor receptor 2; n.s., not significant; SCC, squamous cell carcinoma; WHO, World Health Organization; T, tumor stage; N, lymph node stage; L, lymphatic vessel invasion; V, vein invasion; R, resection boundary

Values are demonstrated in median, if not otherwise indicated

Here is some interesting overview of the significant findings: Laurén classification of the tumor including diffuse, intestinal and mixed was statistically different between the two groups, with the number of patients with intestinal type being higher in the HER2 positive group (*p* = 0.001, Chi-Square Test). Since 20 (34%) of the HER2 positive patients had an initial presentation with advanced disease, we chose 20 patients with HER2 negativity and advanced disease as a control group. Median number and distribution of the metastatic sites were identical in both groups. As a technical issue, 41% and 27% of the patients from HER2 positive and negative groups were evaluated from biopsy specimens, whereas 59% and 73% were resection tissues, respectively. The HER2 positive group comprised more patients with higher gradings as compared to the HER2 negative group (*p* = 0.006, Chi-Square Test). Differences in tumor invasion depth (T), nodal involvement (N), lymph vessel involvement (L) and resection status (R) were not significant between the two groups. Only the involvement of veins (V) was significantly higher in HER2 negative patients (*p* = 0.03, Chi-Square Test).

### Management with Chemotherapy/Antibody Treatment

**Supplementary Table** **1** summarizes the chemotherapy management of the entire group. Sixteen patients (27%) from the HER2 positive group received trastuzumab as part of their treatment regimen. The ratio of patients receiving second-line chemotherapy was significantly higher in the HER2 positive group as compared to the HER2 negative group (22% versus 8%, respectively, *p* = 0.04, Chi-Square Test). However, this ratio was no longer significant in respect of patients receiving third line treatment.

### Staining with PD-L1

Immunohistochemical PD-L1 positivity was assessed in tumor cells (TPS) and tumor associated immune cells (TAIs) separately. Additionally, the combined positive score (CPS) and interface pattern were evaluated [6].

**Fig. 1:**
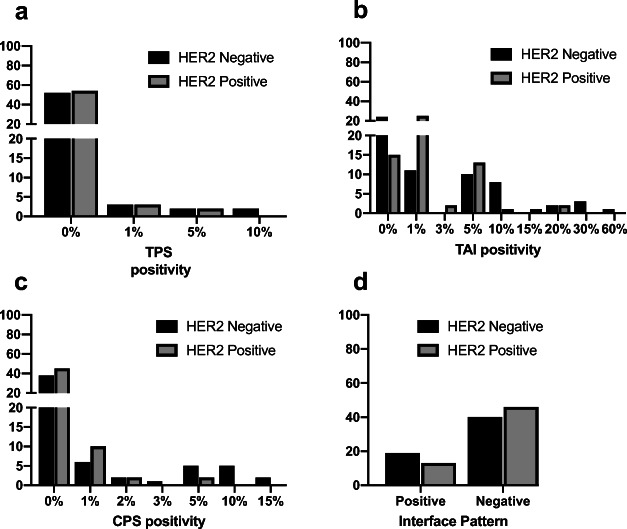
PD-L1 expression levels using different assessments n, number; HER2, human epidermal growth factor receptor 2; TPS, tumor proportion score; TAI, tumor associated immune cells; CPS, combined positive score. Positivity of each assessment (except interface pattern) are demonstrated in continuous variables within X-axis

**Fig. 2:**
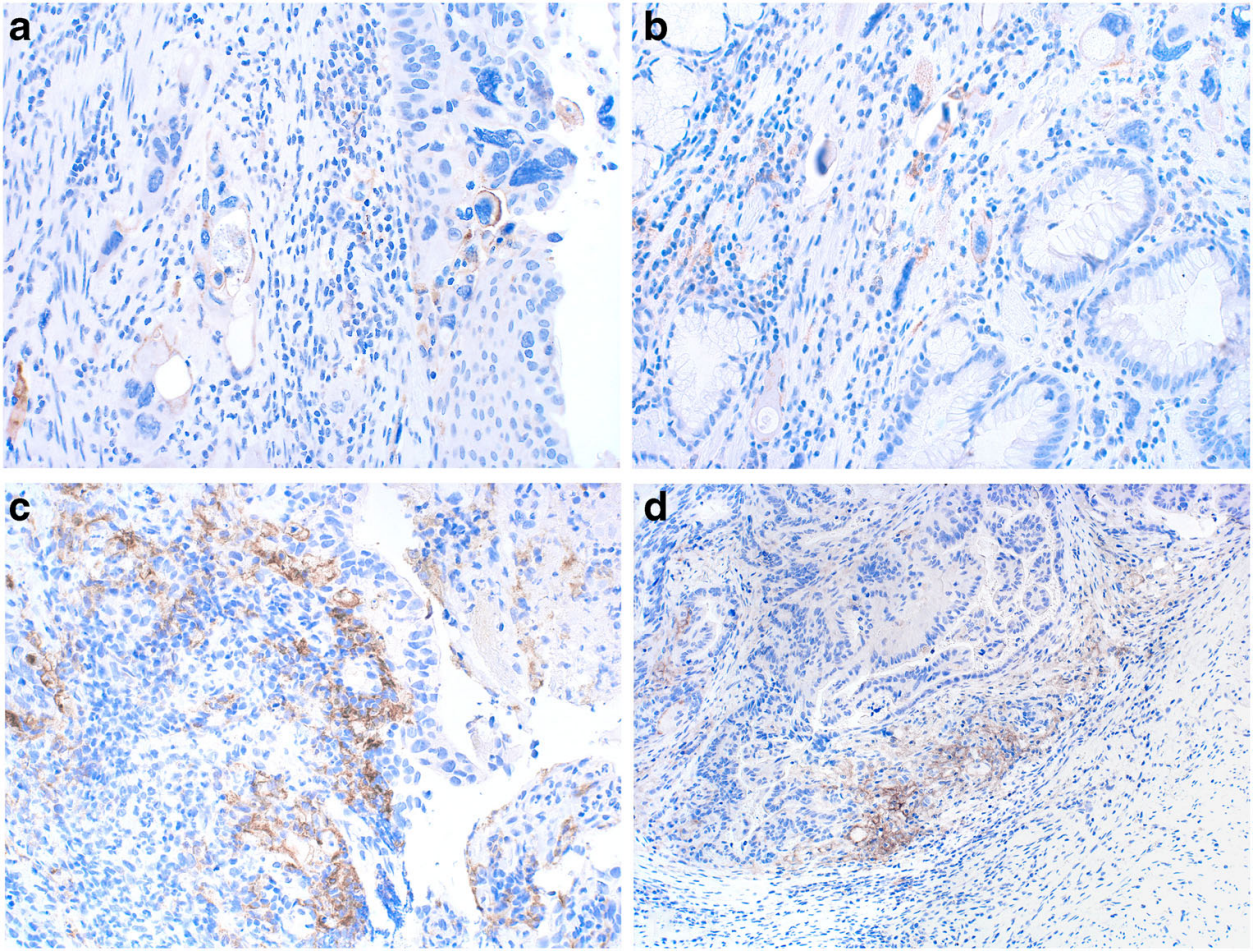
**a** and **b**: Membranous expression (and rarely cytoplasmic staining not used for scoring) of PD-L1 in tumor cells (magnification 200x) **c**: PD-L1 expression of tumor associated immune cells (magnification 200x) **d**: Example of interface pattern. PD-L1-positive immune cells at the interface between carcinoma (top left) and adjacent stroma (magnification 100x)

Nine and 12% of the patients in the HER2 positive and negative group, respectively, showed positivity for at least 1% of PD-L1 in tumor cells (TPS). The difference of PD-L1 positive and negative cases between both groups was not statistically significant **(**Fig. 1**)**. Membranous PD-L1 staining in the tumor cells varied between 0% and 10% with the positive staining pattern being more focal rather than diffuse. For two examples of positive staining of tumor cells see Fig. 2a and b**.**

Seventy-six and 59% of patients in the HER2 positive and negative group, respectively, were positive for at least 1% PD-L1 expression in TAIs **(**Fig. 1**-**b, Fig. 2c**)**. This ratio was again not statistically significant.

Based on the CPS criteria, 25% of the patients in the HER2 positive group and 34% of the HER2 negative group were classified as PD-L1 positive, which again was not statistically significant **(**Fig. 1**-**c**)**.

Interface pattern **(**Figs. 1**-**d and 2d) for PD-L1 staining was evaluated in the entire cohort, where 22% and 32% of the patients were classified to be positive in HER2 positive and negative groups, respectively (not significant).

Furthermore, PD-L1 assessment using different scores was analyzed with regard to the Laurén and WHO classification. TPS, CPS or interface pattern were not associated with any subtype of the Laurén or WHO 2019 classification. However, PD-L1 expression in TAIs was significantly higher in patients with intestinal type (76% versus 24%, *p* = 0.006; Chi-Square Test).

### Survival Analysis

#### Localized Disease

In patients with localized gastroesophageal cancer (*n* = 39 in both groups), overall survival was not significantly different in HER2 positive and negative patients, although there was a tendency towards higher survival rates in HER2 positive patients (median 69 versus 44 months, respectively, log rank test: *p* = 0.4) **(Supplementary Fig.** **1****A)**.

TPS was not associated with the outcome of patients with localized disease (*p* = 0.7, HR = 1; Cox regression), whereas expression of PD-L1 in TAIs was associated with a better outcome (*p* = 0.02, HR = 0.8; Cox regression). Furthermore, PD-L1 scores including CPS also did not influence survival (p = 0.4, HR = 1.1; Cox regression), whereas occurrence of the interface pattern was significantly associated with a better outcome (*p* = 0.04, HR = 0.39; Cox regression). When adjusted for HER2 expression, these survival outcomes did not change.

Further clinical and pathological parameters as well as serum tumor markers analyses were not associated with the outcome. Only initial resection status (R0 or R1) and initial performance status (according to the Eastern Cooperative Oncology Group classification, ECOG) of the patients were significantly associated with the outcome (*p* = 0.01, HR = 3.6; *p* = 0.002, HR = 3.02; respectively).

#### Advanced Disease

In patients with advanced gastro-esophageal cancer (*n* = 20 in both groups), overall survival was significantly different with positive and negative HER2-status, (median 33 versus 16 months, respectively, log rank test = 0.02) **(Supplementary Fig.** **1****B)**.

TPS was associated with an unfavorable outcome in patients with advanced disease (*p* = 0.02, HR = 1.4; Cox regression), whereas expression of PD-L1 in TAIs was not associated with the outcome (*p* = 0.6, HR = 1; Cox regression). Furthermore, PD-L1 scores including CPS did show a significant influence on the outcome with a clinically equal hazard ratio (*p* = 0.03, HR = 1.02; Cox regression), and interface pattern was not associated with the outcome either (*p* = 0.8, HR = 1.1; Cox regression). When adjusted for HER2 expression, these survival outcomes did not change.

Further clinical and pathological parameters as well as serum tumor markers analyses were not associated with the outcome. Interestingly, palliative surgery of the tumor was associated with a better outcome (*p* = 0.04, HR = 0.3; Cox regression).

### Correlation of Different PD-L1 Scores

We performed different scores for the PD-L1 staining, since there does not exist a standardized protocol for the assessment of PD-L1. For this analysis, the quantitative PD-L1 assessment in tumor cells, namely TPS and tumor associated immune cells was translated into dichotomized variables and all patients with ≥1% staining were defined as “positive”. As a result, TPS correlated with CPS (Pearson’s correlations coefficient, 0.46, *p* < 0.001). There was also a positive correlation between TPS and interface pattern (Pearson’s correlations coefficient, 0.48, p < 0.001). CPS also correlated with PD-L1 in TAIs and interface pattern (Pearson’s correlations coefficient, 0.53 vs. 0.6, respectively; p < 0.001 for both).

## Discussion

In the last decade the identification of targeted therapies gained widespread interest in the field of oncology, especially in gastroesophageal cancer. Human epidermal growth factor receptor 2 (HER2) was the only target that showed a clinical benefit in the first-line setting for advanced gastroesophageal adenocarcinoma. The current work retrospectively recruited HER2 positive patients with gastroesophageal cancer and matched these with HER2 negative ones based on histology, location and stage of the disease at the initial onset, either localized or advanced, as well as gender.

Interestingly, the HER2 positive group included significantly more patients with higher gradings than the group of HER2 negative patients. This finding is controversial in view of recent literature, where both poor and well differentiated tumors were found in HER2 positive patients [16]. One possible explanation might be the high number of patients with advanced disease in our population. There is almost a consensus on the distribution of Laurén classification for HER2 positive patients, as the intestinal type was more frequent in this population [16], which was also in line with our findings.

Since our HER2 positive cohort and the control cohort included patients with both localized and advanced setting, the HER2 classification was correlated separately with the outcome in these both settings. Interestingly, HER2 was significantly associated with better outcome in patients with advanced disease, whereas no significant association was observed in the localized group despite a tendency towards higher survival rates for HER2 positive patients. The pivotal ToGA trial tested the anti-HER2 monoclonal antibody trastuzumab in patients with advanced disease, and led to trastuzumab becoming the standard treatment for this setting [9]. However, the role of anti-HER2 treatment in patients with localized disease, in which case perioperative chemotherapy is preferred to date, is not clearly known. An interim analysis of a large phase III trial demonstrated promising complete response rates [24], when trastuzumab was given together with chemotherapy in the perioperative setting. The final data is, however, still expected. It will be interesting to see, whether trastuzumab plays a role in patients with localized disease, as it has in advanced setting [9].

Immunotherapy represents the recent highlight in oncology and has already become the standard treatment in some oncological entities [25]. As suggested by the TCGA classification of gastric cancers, there exists a rationale for a potential response to immunotherapy in this entity based on the expression of PD-L1 in some subgroups. Upon publication of the pivotal phase Ib trial from Muro et al., which demonstrated considerable survival benefits in patients with gastroesophageal cancer under programmed death receptor 1 (PD-1) blockade with pembrolizumab, many clinical trials with different combinations and different settings were initiated [6]. Since HER2 positivity represents an important biological driver marker of the gastroesophageal tumor, HER2 positive patients were for the most part excluded from these clinical trials. In this current report, we sought to shed some light on the potential association of HER2 with PD-L1, which represents one of the major biomarkers of the immune checkpoint inhibitor treatment. The assessment of PD-L1 staining in immunohistochemistry was done based on four different scores: i) PD-L1 expression in tumor cells in terms of tumor proportion score, ii) PD-L1 expression in tumor associated immune cells, iii) combined positive score and iv) PD-L1 expression in interface pattern. In our current report, the distribution of PD-L1 positivity obtained by four different scores was similar in HER2 positive and negative patients. Interestingly, Wang et al. found an association with HER2 negativity and PD-L1 positivity, whereas some other groups such as Oki et al. demonstrated a frequency of PD-L1 in the HER2 positive population [26, 27]. Implementation of anti-HER2 treatment with trastuzumab prolonged the survival of patients with advanced gastroesophageal tumors, however after some time treatment resistance occurred in almost all patients. Anti-HER2 targeting with other drugs was tested as second-line treatment in large trials, which failed to attain promising results [20, 21]. Since PD-L1 distribution is observed to a similar extent in HER2 positive and negative patients, and the survival outcome of PD-L1 was independent of HER2 expression, use of immune checkpoint inhibitors including targeting PD-L1 and PD-1 might bring an additional survival benefit for patients with HER2 positivity. Recent findings from other tumor entities such as hepatocellular carcinoma and renal cell carcinoma indicate, that a targeted therapy and immunotherapy can be safely combined and extend the survival of the patients [28, 29]. Thus these combinations were already approved for those entities. Some promising first in class phase II clinical trials for HER2 positive patients do also show, that the response rates under immune checkpoint inhibitor therapy with pembrolizumab can be dramatically elevated, which might have an impact on the survival [30]. Future and ongoing large clinical trials will answer the question whether addition of immunotherapy to anti-HER2 therapy is beneficial for those patients with HER2 positive gastroesophageal tumors.

The literature reports a marked variation in expression levels of PD-L1 in gastroesophageal cancer. This variation might be due to several very important reasons: i) use of different kinds of antibodies; ii) different kinds of tissues as some studies used tissue microarray blocks, whereas some investigated resected tissues, iii) different cut-off values of PD-L1 expression which are regarded as positive or negative, iv) variety of scores and assessments, and finally v) the patients´ ethnical background. Taking all these factors into account, PD-L1 expression levels in gastroesophageal cancer patients were reported within the range of 14% to 69% [31, 32]. In line with this variation, the current study observed a TPS of 12% and PD-L1 in TAIs of 59% (both for the HER2 negative group). Apart from the percentage of expression of PD-L1 in gastroesophageal cancer, its association with survival outcome shows discordance as well [31, 33]. Gu et al. recently performed a meta-analysis of 15 studies including a total of 3291 patients with gastroesophageal cancer, where PD-L1 expression seemed to be associated with an unfavorable prognosis [34]. This variation in PD-L1 expression and its association with outcome is of particular importance, since these discordances render the potential biomarker capacity of PD-L1 debatable. By way of a typical example, although both pembrolizumab and nivolumab are directed against PD-1, PD-L1 assessment would usually predict the treatment response in pembrolizumab trials, whereas nivolumab trials were biomarker independent [6–8, 35]. It is important to mention that the scoring systems and the antibodies used in these trials were different. Thus, investigating and comparing different antibodies used in different trials in the same run might help to understand the variation underlying the distinct prognostic character of PD-L1. In our current observation, the correlation of different PD-L1 assessments with one another was moderate and sometimes not even present, which again emphasizes the fact that the variation of the PD-L1 assessment is extensive. Notably, PD-L1 expression within a tumor tissue itself might be heterogeneous, which might make a single staining not representative for the whole tissue. Here, alternative or supportive PD-L1 detection methods such as PD-L1 measurements in circulating blood samples might be helpful. Another strategy to overcome difficulties in evaluation of PD-L1 expression might be establishment of other tissue based parameters, which predict response to immunotherapy in a more reliable way. Microsatellite instability (MSI) represents one of the markers with very high predictability for response to immune checkpoint inhibitor therapy. The relation of MSI to HER2 is very interesting, since both large TCGA and Memorial Sloan Catering Cancer Center cohorts of gastroesophageal cancer tumors do show a complete negativity of MSI in HER2 positive tumors, indicating a different driver molecule mechanism of these both markers [2, 36]. These findings might indicate that MSI, despite its high predictive potential for immunotherapy, does not play a major role in HER2 positive patients.

In conclusion, we found extended survival of HER2 positive patients in advanced gastroesophageal cancer, which underlines the importance of identifying subgroups in oncological diseases based on the molecular sub-classification. Expression of PD-L1, a potential biomarker for the immunotherapy response, was observed in HER2 positive and negative patients to a similar extent, and its presence was not influenced by the HER2 status. This might indicate that HER2 positive patients benefit from immune checkpoint inhibitor therapy and therefore should be included in relevant clinical trials. Assessment and scoring of PD-L1 varies in the literature, which indicates that a consistent definition is desperately needed.

## Electronic supplementary material

ESM 1 (DOCX 13 kb)

ESM 2 (PPTX 72 kb)
