# Supplementary material for: PD-L1 and HER2 Expression in Gastroesophageal Cancer: a Matched Case Control Study
Source: Pathol Oncol Res. 2020 May 5;26(4):2225–35. doi: 10.1007/s12253-020-00814-2 (PMC7471145; doi:10.1007/s12253-020-00814-2)
Supplement: Supplementary file 1 — (DOCX 13 kb) [file 12253_2020_814_MOESM1_ESM.docx]

| **Supplementary Table 1: Chemotherapy**  **(CHT) Management** | |  |  |  |
| --- | --- | --- | --- | --- |
|  |  |  |  |  |
|  |  | Her2 positive (n=59) | Her2 negative (n=59) | ***p*** |
|  |  |  |  |  |
| Neoadjuvant CHT (yes/percentage) | | 25 (42%) | 29 (49%) | n.s. |
| Adjuvant CHT (yes/percentage) | | 8 (14%) | 12 (20%) | n.s. |
| Palliative CHT (yes/percentage) | | 26 (44%) | 26 (44%) | n.s. |
| Trastuzumab treatment (yes/percentage) | | 16 (27%) | 1 (2%) | n.s. |
| Trastuzumab maintenance (yes/percentage) | | 7 (12%) | 0 | n.s. |
| Second line CHT (yes/percentage) | | 13 (22%) | 5 (8%) | **0.04** |
| Third line CHT (yes/percentage) | | 6 (10%) | 2 (3%) | n.s. |
|  |  |  |  |  |
