# Supplementary material for: PD-L1 and HER2 Expression in Gastroesophageal Cancer: a Matched Case Control Study
Source: Pathol Oncol Res. 2020 May 5;26(4):2225–35. doi: 10.1007/s12253-020-00814-2 (PMC7471145; doi:10.1007/s12253-020-00814-2)
Supplement: Supplementary file 2 — (PPTX 72 kb) [file 12253_2020_814_MOESM2_ESM.pptx]

## Slide 1
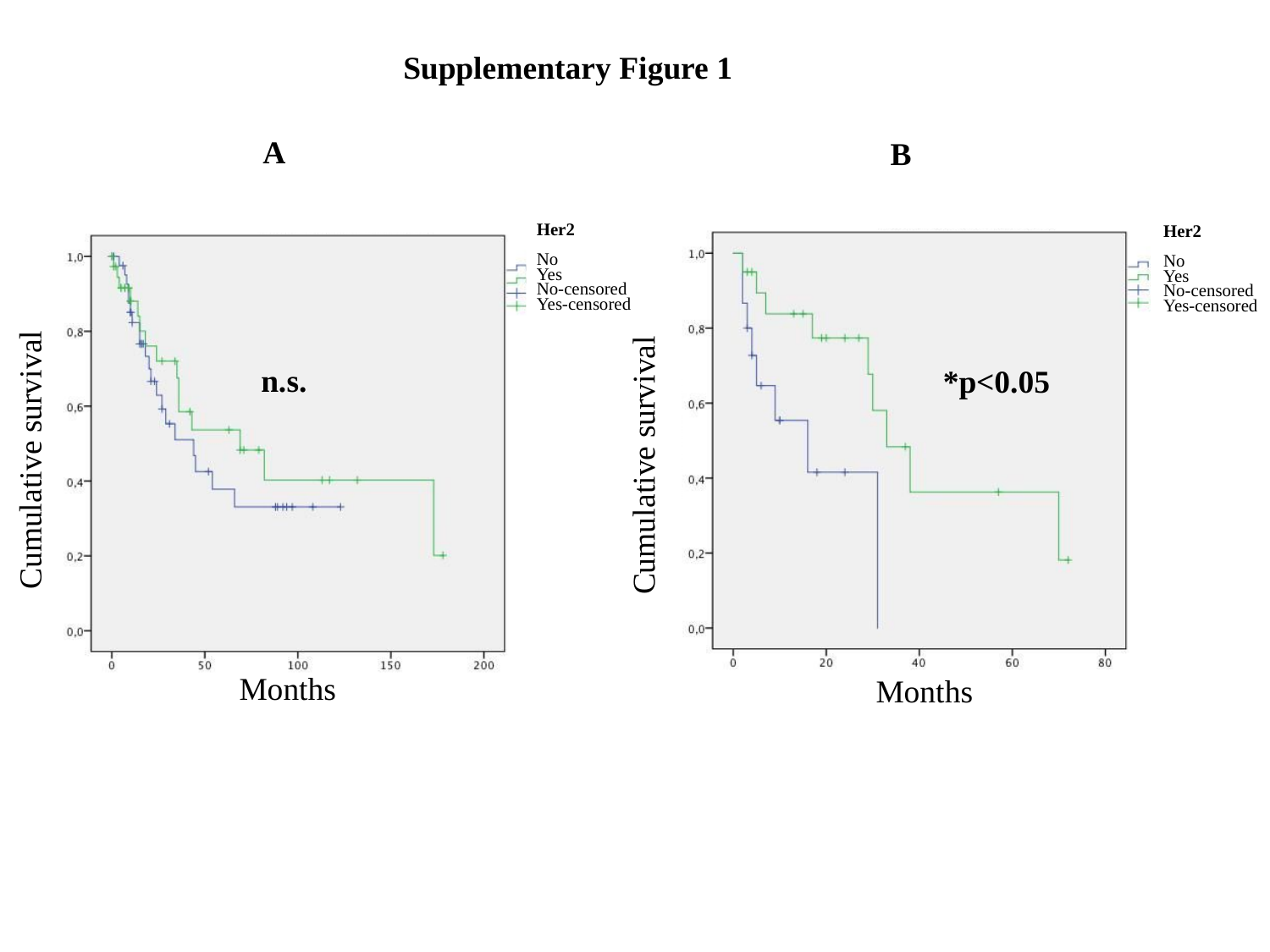

Supplementary Figure 1
A
B
Her2
No
Yes
No-censored
Yes-censored
Her2
No
Yes
No-censored
Yes-censored
n.s.
*p<0.05
Cumulative survival
Cumulative survival
Months
Months
